# Stereoelectroencephalography-guided radiofrequency thermocoagulation

**DOI:** 10.3171/2024.4.FOCVID2442

**Published:** 2024-07-01

**Authors:** Guillaume Dannhoff, Luca Fumagalli, Sarah Ferrand-Sorbets, Georg Dorfmuller, Marion Quirins, Pierre Bourdillon

**Affiliations:** Departments of 1 Neurosurgery,; 2 Pediatric Neurosurgery, and; 3 Neurology, Rothschild Foundation Hospital, Paris, France

**Keywords:** epilepsy surgery, thermocoagulation, stereoelectroencephalography, SEEG

## Abstract

Within the neurosurgeon’s armamentarium, stereoelectroencephalography (SEEG)–guided radiofrequency thermocoagulation (RFTC) is an elegant tool to manage epilepsy in selected cases. This technique can 1) be curative when targeting small-volume ictal onset zones, 2) be used as a diagnostic tool by observing the consequences of coagulation on seizures or by recording the epileptic network in SEEG, and 3) offer palliative treatment through multiple lesions within a wide epileptic network. It is performed on awake patients, under continuous neurological evaluation, while monitoring impedance, time, and energy delivered. It could offer highly favorable outcomes in some cases, as in periventricular nodular heterotopia where 81% of patients are responders.

The video can be found here: https://stream.cadmore.media/r10.3171/2024.4.FOCVID2442

## Transcript

### 0:30 What is SEEG-Guided RFTC?

SEEG-guided radiofrequency thermocoagulation, as known as RFTC, is a lesional stereotactic technique consisting in creating radiofrequency lesions within epileptic networks, via previously implanted SEEG electrodes. The target is selected based on the SEEG recording. This technique is performed at the end of a video-SEEG.

### 0:52 Physical Principles.

During SEEG-guided RFTC, a radiofrequency current is delivered on adjacent contacts of an electrode, thus creating thermal lesions around the electrode. Several lesions can be obtained by coagulating multiple pairs of adjacent contacts, so as to obtain a confluent lesion, as shown here in vitro on albumin.^1,2^

### 1:39 Indications.

There are three main indications of SEEG-guided RFTC: First, this procedure can be curative by destruction of the entire ictal onset zone. This is of particular interest in cases of deep and small-volume ictal onset zones, as they are difficult to access surgically and need to be identified by SEEG. Periventricular nodular heterotopia is the perfect example, as only a small portion of the heterotopia is generally involved in the seizures, thus requiring an intracranial recording to be identified, and as they are typically deeply located lesions. Secondly, SEEG-guided RFTC can be a diagnostic procedure for complex epileptic network. The volume of the RFTC is unlikely to cure the patient but can have a great diagnostic value. Indeed, any improvement—even transient—in frequency or intensity of the seizures has an excellent predictive positive value of surgical success (93%). It is even possible to perform coagulations while the SEEG procedure is still in progress, and to carry on recording to test an electrophysiological hypothesis. Finally, there is a palliative indication in case of wide epileptic networks where multiple lesions can be performed in order to obtain better epileptic control. In such cases, repeated procedures are often necessary to maintain the effect over time.

### 2:54 SEEG Electrodes Implantation.

We can see here the first surgical step, which consists in the implantation of SEEG electrodes, on which coagulation is possible. Following a robot-guided avascular trajectory of implantation, the bone is drilled and the dura coagulated before the insertion of the screw-in base, which the size is adapted to the thickness of bone and the temporalis muscle. We then prepare the electrode path via insertion of a stylus whose length is 5 mm shorter than the exploration length of the electrode. We then introduce smoothly the electrode. It is to be noted that it is not recommended to shave the patient head before an SEEG procedure, the example presented being a patient for whom this is the usual haircut.

### 3:35 Postimplantation Control.

At the end of the procedure, adequate position of the electrodes has to be controlled by a CT scan. We recommend realization of a brain MRI to better evaluate the anatomical position of the electrodes as well as their relationships with adjacent vessels, in order to select potential targets for radiofrequency thermocoagulation.

### 3:52 Contacts Selection.

We can see the early appearance of a low-amplitude fast pattern at the level of the epileptic seizure, considered as either the epileptic focus or a primordial irritative zone that is difficult to access surgically. One or several contacts are selected as candidate for thermocoagulation, after carefully verifying on imaging the absence of vessels in the immediate neighborhood.

### 4:15 Radiofrequency Thermocoagulation.

The patient is awake in the operating room, with permanent clinical monitoring by a neurologist. The electrode chosen to undergo coagulation is connected to the monitor. The radiofrequency thermocoagulation is then performed under continuous clinical evaluation, as our patient can be seen here performing motor and speech tasks under evaluation of the clinician.

### 4:37 Impedance Monitoring.

The increase of energy, which is directly correlated to the power and the voltage delivered, is realized with a maximum time of coagulation. It is necessary to check the impedance throughout the whole process. As SEEG electrodes do not have a thermocouple, it is indeed not possible to directly monitor temperature. These bipolar thermocoagulations are thus monitored through impedance. An abrupt change of impedance means that a coagulation has occurred. Most coagulators will stop automatically in such a case of impedance brutal modification. It is common for patients to perceive a noise, similar to a waterdrop falling on a hot pan, during the coagulation process. Even when this sound is heard, it is appropriate to carry on with the coagulation, as lesion volume can increase past that point.

### 5:24 Postcoagulation Control.

Following the thermocoagulation, electrodes are explanted. When necessary, the explantation can be delayed in order to record the consequences of the coagulation. A postprocedure imaging is finally systematically done after coagulation.

### 5:39 Outcome.

Outcome and efficacy depend on the indication of SEEG-guided RFTC. Current literature depicts periventricular nodular heterotopias as the best indication in terms of results, with 81% responders at 1 year,^3^ and as high as 76% of seizure-free patients.^4,5^

## Disclosures

The authors report no conflict of interest concerning the materials or methods used in this study or the findings specified in this publication.

## Author Contributions

Primary surgeon: Bourdillon. Assistant surgeon: Dannhoff. Editing and drafting the video and abstract: Dannhoff, Fumagalli, Quirins, Bourdillon. Critically revising the work: Fumagalli, Ferrand-Sorbets, Quirins. Reviewed submitted version of the work: Dannhoff, Fumagalli, Ferrand-Sorbets, Dorfmuller, Bourdillon. Approved the final version of the work on behalf of all authors: Dannhoff. Supervision: Dorfmuller, Bourdillon. Neurophysiologist: Quirins.

## Supplemental Material

### Patient Informed Consent

The necessary patient informed consent was obtained in this study.

## References

[1] Bourdillon P Rheims S Catenoix H Surgical techniques: stereoelectroencephalography-guided radiofrequency-thermocoagulation (SEEG-guided RF-TC) Seizure 2020 77 64 68 30711397 10.1016/j.seizure.2019.01.021

[2] Bourdillon P Isnard J Catenoix H Stereo-electro-encephalography-guided radiofrequency thermocoagulation: from in vitro and in vivo data to technical guidelines World Neurosurg 2016 94 73 79 27373418 10.1016/j.wneu.2016.06.095

[3] Bourdillon P Cucherat M Isnard J Stereo-electroencephalography-guided radiofrequency thermocoagulation in patients with focal epilepsy: a systematic review and meta-analysis Epilepsia 2018 59 12 2296 2304 30345535 10.1111/epi.14584

[4] Mirandola L Mai RF Francione S Stereo-EEG: diagnostic and therapeutic tool for periventricular nodular heterotopia epilepsies Epilepsia 2017 58 11 1962 1971 28880999 10.1111/epi.13895

[5] Cossu M Mirandola L Tassi L RF-ablation in periventricular heterotopia-related epilepsy Epilepsy Res 2018 142 121 125 28705474 10.1016/j.eplepsyres.2017.07.001

